# Hematologic and biochemical differences between two free ranging Yangtze finless porpoise populations: The implications of habitat

**DOI:** 10.1371/journal.pone.0188570

**Published:** 2017-11-30

**Authors:** Ghulam Nabi, Yujiang Hao, Xianyuan Zeng, Zheng Jinsong, Richard W. McLaughlin, Ding Wang

**Affiliations:** 1 Institute of Hydrobiology, the Chinese Academy of Sciences, Wuhan, Hubei, PR China; 2 University of the Chinese Academy of Sciences, Shijingshan District, Beijing, PR China; 3 General Studies, Gateway Technical College, Kenosha, Wisconsin, United States of America; Institute of Deep-sea Science and Engineering, Chinese Academy of Sciences, CHINA

## Abstract

The goals of this study were to compare the serum chemistry and hematology values of wild and semi-natural free-ranging Yangtze Finless Porpoises (*Neophocaena asiaeorientalis ssp*. *asiaeorientalis*) populations and to ascertain how these values change with the different environmental condition. For this study, samples were collected from 81 YFPs, 35 living in the wild and 46 living in a semi-natural reserve. Each population was divided into 8 life history categories; Male Calf, Female Calf, Juvenile Male, Juvenile Female, Adult Male, Pregnant, Lactating and Pregnant plus Lactating. Statistically significant differences in the various parameters were observed in the same life history categories for both populations. Generally, Lipid Profile, Hepatic Enzymes, Creatine Kinase, Red Blood Cells, Hemoglobin, Hematocrit and Neutrophils were significantly higher in the Tian-E-Zhou Oxbow population while, Creatinine, Phosphate, Lactate Dehydrogenase, Bilirubin and Lymphocytes were significantly higher in the Poyang Lake YFPs. Across the groups in the Tian-E-Zhou Oxbow population, a significant decrease in serum Albumin, Alkaline Phosphatase and Calcium, while a significant increase in the Neutrophils and Platelets was observed. Similarly, in the Poyang Lake, Alkaline Phosphatase levels in the Female Calves group, High Density Lipoprotein Cholesterol in Lactating group, basophil counts in Pregnant plus Lactating group, lymphocytes counts in Juvenile Females group and Globulin and Total Protein levels in Pregnant group were significantly higher. This study in health assessments can help us to understand the effect of sex, age, reproductive status and environmental conditions on the well-being of Yangtze Finless Porpoises.

## Introduction

Worldwide approximately more than an estimated 10,000 vertebrates are considered threatened species. Deleterious alternations in habitat quality are potential threats to worldwide biodiversity [1,2]. In spite of serious habitat conservation measures, loss of habitat, especially in biodiversity rich hotspots around the world, is expected to cause mass extinction, especially for endemic and threatened species [3]. Therefore, investigating in the way in which anthropogenic alterations to habitat quality affects a population is essential to the preservation of an individual species and biodiversity [4]. Habitat selection can affect the fitness and physiology of an organism by changing the availability of resources to the animals [5]. The physiology of the animal is affected by environmental conditions. There is an essential connection between the availability of prey species and the population growth rate [6]. It may also show a mechanistic approach to the understanding why and how organisms response and biological processes involved [5,7,8]. Furthermore, physiology is essential for the identification of sink habitats [9], population responses to altered habitats and determining the causes of population declines [8,10]. In vertebrates, physiological indices such as, body growth, fat reserves, hematology counts, blood chemistry, chemistry profiles of urine, heat shock proteins and adrenocortical hypertrophy have been used to rank habitat quality [11,12]. Predicting habitat quality alterations on the basis of individual physiological responses can be a useful conservation tool [7]. Using this capability, conditions for vertebrate populations can be improved either by avoiding poor quality habitats or by modifying current management practices [13].

Understanding the population responses of the Yangtze finless porpoise (YFP) (*Neophocaena asiaeorientalis ssp*. *asiaeorientalis*) to altered habitat is of particular interest, because of their exposure to a variety of industrial pollutants [14–16], agricultures run offs [15] and toxins [15,17–19]. They are also exposed to severe acoustic pollution from dynamite explosions [20] and vessels noise [15,18,21]. In addition, oil spills from boats causes further habitat degradation [15]. YFPs are currently classified as a critically endangered species by the IUCN Red List of Threatened Species [22]. There are only 1040 individuals left in the world [23]. According to the prediction of Mei et al. (2012), YFPs in the wild could become extinct within the next 63 years. This value is based on the life table of stranded carcasses collected between 1994 and 2008 [24]. There are approximately 450 YFPs living in the Poyang Lake [23], the largest freshwater lake in China. In 1992, the Chinese Government selected the Tian-E-Zhou Oxbow, located in the middle region of the Yangtze River, as a natural *ex situ* reserve for the protection of YFPs. Both the ecological and environmental conditions of this oxbow are identical to those of the Yangtze River since both waterways are connected. According to a census conducted in 2015 at the Tian-E-Zhou Oxbow, the porpoise population increased from 25 individuals as determine in 2010 to over 60 YFPs [25]. To date, no physiological measurements have been made on YFPs living in the wild or in semi-natural reserves in order to evaluate habitat quality. The objective of this study, therefore, was to evaluate and compare the habitat quality of two apparently healthy free ranging YFPs populations living in the wild or in semi-natural reserves, using hematology and biochemistry parameters. This work will aid in supporting risk assessment of porpoise populations, in improving habitat conditions and in adaptive management practices.

## Materials and methods

### Animal ethics

All appropriate ethics and protocol approvals were obtained for this research from the Ministry of Agriculture of the People’s Republic of China. The blood sampling procedure was also reviewed and approved by the Research Ethic Committee of Institute of Hydrobiology, Chinese Academy of Science. In addition, this entire study strictly adhered to Chinese law and ethical guidelines for wild animals. No anesthesia, euthanasia, or any other kind of surgical procedure was used in this study.

### Study location

In China, Poyang Lake is the largest freshwater lake, located in the Jiangxi Province on the southern bank and the middle reaches of the Yangtze River. It is located at longitude 115° 47′- 116° 45′ east and latitude 28° 22′- 29° 45′ north. It covers a total area of 1.6×10^5^ km^2^, occupying about 96.85% of the Jiangxi province land mass, which accounts for approximately 9% of the Yangtze River basin. Seasonal changes occur in the size of the lake. In summer, its size exceeds a maximum area of 4000 km^2^ but, in winter and fall, its size decreases to less than 3000 km^2.^ This lake receives water primarily from the Xinjiang River, Raohe River, Fuhe River, Xiushui River and Ganjiang River. Water is then emptied into the Yangtze River [26–28]. The average water depth of the lake is 8.4 m (maximum depth 25.1 m) [26]. In Poyang Lake, there is intensive shipping, dredging, dynamite explosions, acoustic pollution, overfishing, and use of harmful and illegal fishing tool, oil spills from motorboats, and the introduction of various toxins [15, 19, 20]. All of these factors result in a higher mortality for YFPs.

Tian-E-Zhou reserve (E11 2° 31′—112° 36′, N29° 46 ′—29° 51′) is a semi-natural oxbow shape lake and it is an ideal habitat for YFPs. It is located on the north bank of the Yangtze River in Shishou city, Hubei Province. This lake is about 21 km long and 1–2 km wide. In 1972, through natural events the water flow changed so it no longer connected to the Yangtze River [29,30]. In this oxbow, there is no intensive shipping, dredging is not allowed, and fishing is banned over a certain period every year. However, the water is polluted due to nearby farmland drainage. This reserve is regularly managed and involves the regular capture of YFPs for both physical and medical examinations as well as research purposes [31].

### Study design

A total of 81 YFPs were sampled for the study from Poyang Lake (n = 35) and Tian-E-Zhou Oxbow (n = 46). YFPs from Poyang Lake were sampled in March 2015 and YFPs from Tian-E-Zhou Oxbow were sampled in November 2015. This was done during a routine capture release operation. Each population was divided into 8 life history categories; male calf (MC), female calf (FC), juvenile male (JM), juvenile female (JF), adult male (AM), adult female (AF), pregnant (P), lactating (L) and pregnant plus lactating (PL). The calf, juvenile and adult states were categorized on the basis of body length [32]. Pregnancy was confirmed by ultrasound (LOGIQ Book XP, New York, America) examination of the reproductive tract, while lactation state was categorized by the presence of milk. All the detailed information about the study populations are summarized in Table 1.

**Table 1:** Basic information of life history variables for the studied population of ranging Yangtze finless porpoises in Tina-E-Zhou Oxbow and Poyang Lake. [BL = body length (cm); BW = body weight (Kg)].

Tina-E-Zhou Oxbow					Poyang Lake				
ID	BL	BW	BW/BL	Status	ID/PIT	BL	BW	BW/BL	Status
TEZ M26	93.25	19.3	0.206	MC	3351	108	30.3	0.280	MC
TEZ M23	106.5	20.6	0.193	MC	3361	101	20.8	0.205	FC
TEZ F15	104.75	24.6	0.234	FC	3310	103	29.8	0.289	FC
TEZ F08	106	24.4	0.230	FC	3391	105	29.5	0.280	FC
TEZ M24	113	27.9	0.246	JM	3375	108	29.6	0.274	FC
TEZ M01	111	24.9	0.224	JM	3305	107	31.3	0.292	FC
TEZ M06	119	27.8	0.233	JM	3349	122	36.8	0.301	JM
TEZ M07	125	31.4	0.251	JM	3392	124	24	0.193	JM
TEZ M18	133	35.2	0.264	JM	3377	112	31.8	0.283	JM
TEZ M19	127	31.5	0.248	JM	3394	129	35.7	0.276	JM
TEZ M28	125.5	34.3	0.273	JM	3325	129	37.6	0.291	JM
TEZ F11	111	23	0.207	JF	PYF3	120	28	0.233	JF
TEZ F17	116.5	28.2	0.242	JF	3323	115	34.7	0.301	JF
TEZ F12	121	29.8	0.246	JF	3341	123	40.3	0.327	JF
TEZ F04	122	28	0.229	JF	3368	125	36.3	0.290	JF
TEZ F05	125	38.8	0.310	JF	3337	127	45.7	0.359	JF
TEZ M22	140.5	35.3	0.251	AM	3364	138	46.8	0.339	AM
TEZ M04	145	45.3	0.312	AM	3326	144	53.1	0.368	AM
TEZ M05	141	36.8	0.260	AM	3389	145	49.8	0.343	AM
TEZ M08	148	40.8	0.275	AM	3302	152	53.8	0.353	AM
TEZ M12	150.6	44.5	0.295	AM	3319	153	54.4	0.355	AM
TEZ M13	153	47.8	0.312	AM	3355	159.5	69.8	0.437	AM
TEZ M14	152.5	46.3	0.303	AM	3301	154	51.2	0.332	AM
TEZ M10	153.5	50.2	0.327	AM	3356	160	57.2	0.357	AM
TEZ M17	155.75	46.1	0.295	AM	3340	134	60.2	0.449	P
TEZ M09	160	NA	NA	AM	3381	138	67.5	0.489	P
TEZ M27	161	NA	NA	AM	3327	148	NA	NA	P
TEZ M15	163.2	52.7	0.322	AM	3315	131	42.9	0.327	L
TEZ M16	163.5	59.3	0.362	AM	3348	136	41.1	0.302	L
TEZ M11	167.5	62.3	0.371	AM	3316	147	52.9	0.359	L
TEZ M25	NA	40.2	NA	AM	3318	149	57.1	0.383	L
TEZ F25	139.25	45.8	0.328	P	3330	145	58.5	0.403	L
TEZ F19	143	55.3	0.386	P	PYF16	158	56.3	0.356	L
TEZ F21	147.5	53.4	0.362	P	3345	134	58.7	0.438	PL
TEZ F18	149.5	51.1	0.341	P	3329	147	72.1	0.490	PL
TEZ F06	150.6	51.6	0.342	L					
TEZ F26	141	45.6	0.323	L					
TEZ F23	137.5	36.9	0.268	L					
TEZ F24	141.5	42.5	0.300	L					
TEZ F16	152.6	49.4	0.323	L					
TEZ F03	153	39.9	0.260	L					
TEZ F09	160.5	46.7	0.290	L					
TEZ F01	137	48.9	0.356	PL					
TEZ F22	141	40.6	0.287	PL					
TEZ F13	146	50	0.342	PL					
TEZ F14	137	40.5	0.295	PL					

### Animals catching, handling and blood sampling

The animals living in Poyang Lake or in the Tian-E-Zhou Oxbow were captured by using a method called “sound chase and net capture” [33]. Briefly, the animals were chased by several parallel fishing boats with the noise of their engines. The boat speed was usually lower than 10 km/h. When the animals swam into the area where two net boats waiting, the nets were released making an enclosed circle around the animals. After gradually reducing the enclosed area, the animals were taken out of the water and transported to the medical boat for a physical examination. The animals were put on a sponge mattress and were gently restrained in place. Blood samples from each YFP of both populations were obtained within 30 minutes after catching, using a 10 ml disposable syringe (Gemtier, G/Ø/L: 21/0.7/31 mm, 201502, Shanghai, China). All the blood samples were obtained aseptically from the dorsal main vein of the tail fluke. 1ml of blood was transferred to heparinized tubes (Nihon, 161–8560, Tokyo, Japan) for hematology. The remaining samples were centrifuged (Eppendorf AG, 22332, Hamburg, Germany) for 15 min at 1500×g and the serum obtained was transferred to frost-free plastic tubes and stored at -25°C. During the whole process, breath frequency and the behavioral reaction of the animals were monitored. Water was continuously poured on the animal to avoid skin dehydration. The whole examination for one animal usually took less than 15min. All the animals were then gently released after attaching electronic tags.

### Laboratory analysis

Complete blood count; Red Blood Cells (RBCs), Haemoglobin (Hb), Haematocrit (HCT), Mean Corpuscular Haemoglobin (MCH), Mean Corpuscular Haemoglobin Concentration (MCHC), Mean Corpuscular Volume (MCV), Platelets (PLT), Mean Platelet Volume (MPV), Platelet Crit (PCT), Platelet Distribution Width (PDW), Red Cell Distribution Width (RDW) White Blood Cells (WBCs), Neutrophils, Lymphocytes, Monocytes, Basophils and Eosinophils were analysed by using the hematology analyzer (Beckman-Coulter, DxH 800, Porto, Portugal) according to the manufacturer instruction. Liver function parameters; Alaline amino Transferase (ALT), Aspartate amino Transferase (AST), Alkaline Phosphatase (ALP), Gamma-glutamyl Transferase (GGT), Total Bilirubin (T-BILI), Direct Bilirubin (D-BILI), Indirect Bilirubin (I-BILI), Total Bilirubin b (TBILb), Direct Bilirubin b (DBILb; Total Bile Acid (TBA), lipid profile; (Total Cholesterol (TC), Triglyceride (TG), High Density Lipoprotein cholesterol (HDL-c), Ligh Density Lipoprotein cholesterol (LDL-c), enzymes; (lipase, Amylase (AMS), Creatine Kinase (CK), Lactate Dehydrogenase (LDH), Electrolytes (K^+^, Na^+^, Cl^-^, Ca^2+,^ PO4^3−^) and other biochemical parameters such as Total Protein (TP), Albumin (AlB), Globulin (GLB), Glucose (GLU), Blood Urea Nitrogen (BUN), Carbon Dioxide (CO_2_), Urea (UA) and Creatinine (Cr) were measured using the automated clinical chemistry analyzer (Beckman-Coulter, AU5400, Porto, Portugal). The analyzer was calibrated before each assay.

### Statistical analysis

An unpaired Student’s t-test was used to compare one group from one population to its respective group from another population using Graph Pad Prism, *version 5*.*01* (*Graph Pad Software Inc*., San Diego, CA, USA). A one-way ANOVA followed by the post hoc test was used to investigate changes in the mean level of each parameter across all groups for each population. Normality was assessed using the Shapiro-Wilk test. In some groups, due to small sample size, Mann Whitney U test was additionally conducted on the significant parameters to further evaluate their relevance. All data were presented as mean ± SEM. Significance was accepted at p < 0.05.

## Results

### Female calves (FC)

The results of all the biochemical and hematological parameters are summarized in Tables 2 and 3. FC living in the Tian-E-Zhou Oxbow showed statistically significantly higher serum concentration of TC, lipase, AST, ALT, GGT, CK, ALB and BUN. In Poyang Lake YFPs, only serum P and AMS were statistically significantly higher. Blood parameters like RBCs, Hb, HCT and MCV were statistically significantly higher in YFPs living in the Tian-E-Zhou Oxbow compared to YFPs living in Poyang Lake.

**Table 2:** Biochemical parameters of ontogenetic groups.

Parameters	Juvenile Male (Mean±SEM)	Juvenile Female (Mean±SEM)	Female Calf (Mean±SEM)	Adult Male (Mean±SEM)
**ALT** (U/L)	^a^28.62±5.38*^b^9.90±1.07	^a^39.50±10.87***^b^ 10.28 ±1.40	^a^51.25±19.05**^b^9.09±1.59	^a^31.77±1.79***^b^17.39±3.22
**AST**(U/L)	^a^214.9±15.90**^b^131.9±13.72	^a^ 258.9±58.86*^b^ 154.3 ±7.76	^a^329.0±101.0**^b^139.3±4.09	^a^200.8±8.23***^b^147.2±9.71
**T-BILI**(μmol/L)	^a^3.13±0.36^b^3.37±0.38	^a^ 2.500 ±0.47^b^ 3.35 ±0.19*	^a^2.20±0.60^b^3.26±0.33	^a^3.28±0.10^b^6.17±2.34
**D-BILI**(μmol/L)	^a^1.10±0.10^b^0.80±0.32	^a^ 0.90±0.10^b^ 1.022 ±0.06	^a^0.80±0.20^b^1.00±0.10	^a^0.86±0.07^b^1.58±0.54*
**I-BILI**(μmol/L)		^a^1.80±0.90^b^2.42±0.17		
**TBILb**(μmol/L)	^a^50.78±12.78^b^65.20±6.70	^a^ 72.60 ±21.88^b^ 66.43 ±11.32	^a^100.1±24.00^b^79.04±18.17	^a^31.97±10.36^b^235.5±91.01**
**DBILb**(μmol/L)	^a^35.72±15.52^b^23.33±5.85	^a^ 34.53 ±16.80^b^ 19.36 ±4.03	^a^27.05±21.45^b^19.58±7.06	^a^18.15±7.15^b^112.9±40.38**
**TP**(g/L)	^a^78.34±2.69*^b^70.35±2.06	^a^ 80.88 ±4.30^b^ 74.94 ±1.92	^a^78.55±7.45^b^71.80±2.22	^a^81.93±1.70^b^81.60±3.02
**AlB**(g/L)	^a^48.45±1.24*^b^44.15±1.59	^a^51.83 ±1.79*^b^ 47.42 ±1.06	^a^53.75±3.35*^b^47.38±1.46	^a^48.52±0.78^b^47.13±1.93
**GLB**(g/L)		^a^33.30±5.90^b^28.90±4.21	^a^20.85±0.15^b^24.42±1.43	
**GGT**(U/L)	^a^31.43±2.79^b^25.73±3.16	^a^ 37.28 ±6.48^b^ 29.06 ±1.57	^a^34.55±0.65*^b^27.90±1.22	^a^34.62±2.24^b^40.99±6.92
**ALP**(U/L)	^a^230.7±30.85^b^342.8±131.6	^a^ 460.1 ±128.1^b^ 571.1 ±115.2	^a^594.7±241.5^b^696.0±145.9	^a^107.3±12.41^b^106.6±14.88
**TBA**(μmol/L)	^a^5.23±0.99^b^3.45±0.56	^a^ 8.675 ±3.34^b^ 4.822 ±0.57	^a^5.70±2.00^b^4.02±0.74	^a^5.96±0.66^b^5.95±1.25
**GLU**(mmol/L)	^a^6.68±0.22^b^7.51±0.23*	^a^ 7.410 ±0.50^b^ 7.797 ±0.98	^a^7.53±0.53^b^8.63±1.76	^a^7.15±0.33^b^7.30±0.38
**BUN**(mmol/L)	^a^17.77±1.45^b^18.06±0.71	^a^ 20.33 ±0.66*^b^ 17.72 ±0.70	^a^21.31±0.84**^b^16.75±0.39	^a^16.40±0.79^b^17.86±0.95
**CO_2_**(mmol/L)	^a^20.57±1.79^b^18.73±1.30	^a^ 22.33 ±2.35^b^ 19.68 ±0.93	^a^23.85±0.65^b^19.92±1.69	^a^20.60±1.76^b^18.35±1.26
**UA**(μmol/L)	^a^47.52±9.44^b^39.78±7.24	^a^ 56.05 ±5.68*^b^ 40.12 ±3.87	^a^54.00±13.30^b^37.36±4.97	^a^34.41±4.50^b^44.91±8.90
**TC**(mmol/L)	^a^6.68±0.49**^b^3.00±1.11	^a^6.360 ±0.37***^b^3.868 ±0.34	^a^5.94±0.54*^b^3.66±0.47	^a^6.22±0.33***^b^3.93±0.57
**TG**(mmol/L)	^a^1.02±0.17^b^0.73±0.18	^a^1.023 ±0.18^b^0.9711 ±0.12	^a^0.95±0.36^b^0.99±0.18	^a^0.96±.09^b^1.52±0.34*
**HDL-c**(mmol/L)	^a^2.80±0.16***^b^0.93±0.25	^a^2.570 ±0.10*^b^1.776 ±0.18	^a^2.61±0.08^b^1.68±0.29	^a^2.68±0.08***^b^1.61±0.25
**LDL-c**(mmol/L)	^a^0.52±0.05^b^0.66±0.35	^a^0.4675 ±0.042^b^0.4089 ±0.058	^a^0.40±0.05^b^0.42±0.06	^a^0.50±0.04^b^1.11±0.34*
**CK**(U/L)	^a^141.5±38.04*^b^17.35±2.55	^a^145.0 ±24.10***^b^52.13 ±5.03	^a^171.4±45.55**^b^60.48±5.73	^a^80.33±6.76***^b^36.85±9.53
**LDH**(U/L)	^a^105.0±25.36^b^262.6±39.86**	^a^134.3 ±24.04^b^278.4 ±48.51*	^a^108.1±22.35^b^236.7±60.78	^a^113.9±13.74^b^275.4±26.07***
**AMS**(U/L)	^a^15.10±3.51^b^17.78±8.14	^a^16.03 ±4.64^b^21.91 ±1.61	^a^12.60±0.10^b^20.70±0.44***	^a^20.78±1.93^b^53.00±18.73*
**Lipase**(U/L)	^a^11.15±0.36*^b^9.97±0.18	^a^13.53 ±1.35**^b^ 10.19 ±0.24	^a^13.00±1.10**^b^10.08±0.35	^a^11.04±0.20^b^10.69±0.44
**Cr**(μmol/L)	^a^48.28±2.89^b^73.78±10.31*	^a^56.85 ±5.94^b^72.14 ±3.91*	^a^59.40±14.10^b^66.98±3.15	^a^52.99±4.26^b^78.53±4.31***
**K**(mmol/L)	^a^4.89±0.26*^b^4.13±0.25	^a^4.725 ±0.33^b^4.363 ±0.18	^a^4.68±0.80^b^4.19±0.25	^a^4.25±0.13^b^4.21±0.16
**Na**(mmol/L)	^a^157.9±2.32^b^156.7±3.42	^a^158.1 ±2.13^b^158.7 ±1.27	^a^160.2±1.45^b^157.6±2.27	^a^156.4±0.79^b^154.6±3.77
**Cl**(mmol/L)	^a^110.2±3.47^b^108.8±1.84	^a^105.7 ±1.26^b^ 106.7 ±0.69	^a^106.5±2.80^b^106.4±1.08	^a^106.5±1.37^b^104.8±2.23
**Ca**(mmol/L)	^a^2.58±0.05^b^2.52±0.04	^a^ 2.753 ±0.01^b^ 2.702 ±0.04	^a^2.75±0.03^b^2.70±0.07	^a^2.52±0.03^b^2.61±0.06
**P**(mmol/L)	^a^1.07±0.19^b^1.61±0.17*	^a^1.383 ±0.05^b^1.779 ±0.07**	^a^1.30±0.08^b^1.83±0.10*	^a^1.05±0.10^b^1.64±0.04***

^(a)^ = Tina-E-Zhou Population,

^(b)^ = Poyang Lake population.

Significant difference with *P<0.05, **P<0.01 and ***P<0.0001

**Table 3:** Hematologic parameters of ontogenetic groups.

Parameters	Juvenile Male (Mean±SEM)	Juvenile Female (Mean±SEM)	Female Calf (Mean±SEM)	Adult Male (Mean±SEM)
**RBCs**(x 10^12^/L)	^a^5.37±0.20*^b^4.38±0.49	^a^5.38±0.12^b^4.87±0.20	^a^5.25±0.18*^b^4.61±0.16	5.18±0.11*4.89±0.07
**Hb**(g/L)	^a^168.7±6.15^b^143.8±16.32	^a^177.0±3.51*^b^142.8±8.59	^a^166.5±1.50*^b^142.6±5.32	170.8±3.12**155.8±2.85
**HCT**(%)	^a^54.78±2.11**^b^41.04±4.52	^a^57.33±0.78***^b^44.63±0.61	^a^54.20±0.20**^b^42.50±1.13	55.47±1.00***45.25±0.73
**MCH**(pg)	^a^31.38±0.22^b^32.84±0.22***	^a^32.87±0.52^b^29.34±1.37	^a^31.70±0.80^b^31.10±1.77	33.01±0.5031.81±0.38
**MCHC**(g/L)	^a^308.2±2.50^b^349.8±2.57***	^a^309.0±2.00^b^316.2±19.12	^a^307.0±2.00^b^329.6±16.54	308.0±1.04344.3±1.41***
**MCV**(fL)	^a^101.9±0.97***^b^93.90±1.05	^a^106.5±1.74**^b^93.16±1.81	^a^103.4±3.20*^b^94.32±1.68	107.2±1.66***92.41±1.00
**PLT**(x 10^9^/L)	^a^138.7±8.19^b^143.0±11.03	^a^161.7±10.68^b^165.0±21.15	^a^173.0±17.00^b^158.0±31.93	145.4±8.68142.9±8.34
**MPV**(fL)	^a^11.82±0.21^b^11.74±0.16	^a^10.60±0.56^b^12.56±0.65*	^a^11.05±1.25^b^12.36±0.54	11.35±0.1311.73±0.21
**PCT**(%)	^a^0.16±0.00^b^0.16±0.01	^a^0.17±0.00^b^0.16±0.02	^a^0.18±0.00^b^0.15±0.01	0.16±0.000.16±0.01
**PDW**10 (GSD)	^a^16.42±0.15^b^16.14±0.15	^a^16.37±0.18^b^17.44±0.67	^a^16.35±0.35^b^16.60±0.58	16.28±0.1116.05±0.05
**RDW**(% cv)	^a^53.72±1.05^b^55.20±1.83	^a^54.23±0.96*^b^49.86±1.02	^a^53.50±0.80^b^54.34±1.65	12.97±0.1513.29±0.15
**WBCs**(x 10^9^/L)	^a^8.38±0.48^b^9.66±2.53	^a^8.09±1.10^b^8.64±0.51	^a^8.38±0.59^b^8.26±0.84	6.12±0.376.10±0.30
**Neutrophil**(%)	^a^68.48±2.59***^b^42.85±1.35	^a^56.87±5.12^b^46.63±5.45	^a^63.80±5.50^b^63.85±3.65	72.68±2.14***57.44±3.31
**Lymphocyte**(%)	^a^26.50±2.44^b^43.15±5.35**	^a^39.67±6.24^b^42.40±3.10	^a^33.20±5.10^b^39.03±5.71	20.37±1.727.80±2.95*
**Monocyte**(%)	^a^1.81±0.21^b^1.55±0.35	^a^1.16±0.26^b^1.20±0.40	^a^1.75±0.05^b^1.75±0.35	1.40±0.211.28±0.16
**Basophil**(%)	^a^0.05±0.02^b^0.15±0.05*	^a^0.06±0.03^b^0.07±0.02	^a^0.05±0.05^b^0.20±0.10	0.08±0.010.08±0.02
**Eosinophil**(%)	^a^3.15±0.96^b^12.30±3.60**	^a^2.23±0.96^b^11.60±5.12	^a^1.20±0.40^b^3.56±2.56	5.46±1.1713.39±1.58***

^(a)^ = Tina-E-Zhou Population,

^(b)^ = Poyang Lake population.

Significant difference with *P<0.05, **P<0.01 and ***P<0.0001.

### Juvenile males (JM)

For JM living in the Tian-E-Zhou Oxbow, the lipid profile (TC, HDL-c), certain enzymes (ALT, AST, lipase, CK), electrolyte (K) and other biochemical parameters (ALB and TP) were statistically significantly higher versus JM living in Poyang Lake (Table 2). However, for JM living in Poyang Lake, serum LDH, Cr, glucose and P levels were statistically significantly higher compared to the porpoises living in the Tian-E-Zhou Oxbow. Hematological analysis revealed significantly higher levels of RBCs, HCT, MCV and neutrophils in YFPs living in the Tian-E-Zhou Oxbow while significantly higher levels of MCH, MCHC, lymphocytes, basophils and eosinophil were observed in porpoises living in Poyang Lake (Table 3).

### Juvenile females (JF)

Similarly to JM, the lipid profile of JF living in the Tian-E-Zhou Oxbow also showed statistically significantly higher serum concentrations of TC, HDL-c and lipase. Hepatic enzymes such as ALT, AST and other parameters such as ALB, BUN, UA and CK were also statistically significantly elevated in JF YFPs living in the Tian-E-Zhou Oxbow. For YFPs living in Poyang Lake, only LDH, TBILI, Cr and P levels were statistically significantly higher (Table 2). For porpoises living in the Tian-E-Zhou Oxbow, Hb, HCT, MCV and RDW were statistically significantly higher while in Poyang Lake, only MPV was statistically significantly higher for porpoises living in Poyang Lake (Table 3).

### Adult males (AM)

Similarly to the JM and JF, AM porpoises living in the Tian-E-Zhou Oxbow had statistically significantly higher serum levels of TC and HDL-c. The only exception was the levels of LDL-c and TG which were significantly higher in animals living in Poyang Lake. Similarly, hepatic enzymes such as AST, ALT as well as the enzyme CK were also statistically significantly elevated in AM living in the Tian-E-Zhou Oxbow. However, serum AMS, Cr, DBILI, DBILb, TBILb, LDH and P levels were statistically significantly higher in AM living in the Tian-E-Zhou Oxbow (Table 2). YFPs living at the Tian-E-Zhou Oxbow had statistically significantly higher levels of RBCs, Hb, HCT, MCV and neutrophils, while YFPs living in Poyang Lake showed significantly higher levels of MCHC, lymphocytes and eosinophils (Table 3).

### Pregnant females (P)

The same as documented in other groups, HDL-c was statistically significantly higher in PF living in the Tian-E-Zhou Oxbow. Other parameters such as CO_2_ and TBA levels were statistically significantly elevated in PF living in the Tian-E-Zhou Oxbow. In porpoises living in Poyang Lake, the serum level of ALB, Ca, P, TP, DBILb and TBILb were statistically significantly higher (Table 4). PF living in the Tina-E-Zhou Oxbow showed statistically significantly higher RBCs, Hb and MCV counts, while MCHC, WBCs (log transformed data) and monocytes counts were statistically significantly higher in porpoises living in Poyang Lake (Table 5).

**Table 4:** Biochemical parameters of reproductive states groups.

Parameters	Pregnant (Mean±SEM)	Lactating (Mean±SEM)	Pregnant + lactating (Mean±SEM)
**ALT**(U/L)	^a^19.35±2.03^b^25.20±4.19	^a^46.38±10.34**^b^16.18±2.61	^a^36.70±14.13^b^12.95±7.15
**AST**(U/L)	^a^162.6±9.50^b^139.7±9.58	^a^203.7±21.62*^b^141.4±8.86	^a^188.2±13.77^b^172.1±37.40
**T-BILI**(μmol/L)	^a^3.25±0.12^b^4.73±1.67	^a^2.88±0.28^b^4.38±0.62*	^a^1.05±0.08^b^1.97±0.38*^log^
**D-BILI**(μmol/L)	^a^0.97±0.08^b^1.33±0.28	^a^0.86±0.06^b^1.28±0.47	^a^0.63±0.16^b^1.05±0.75
**TBILb**(μmol/L)	^a^16.88±5.04^b^76.20±28.36*	^a^60.28±21.66^b^160.1±67.84	^a^36.90±16.68^b^355.7±9.45***
**DBILb**(μmol/L)	^a^8.07±4.44^b^43.50±14.46*	^a^17.20±3.01^b^48.43±25.0	^a^12.50±7.76^b^108.6±106.0**
**TP**(g/L)	^a^74.80±1.09^b^93.57±6.63*	^a^81.93±2.20^b^78.10±4.20	^a^77.27±1.53^b^77.05±4.95
**AlB**(g/L)	^a^44.65±1.73^b^51.13±2.78*	^a^45.18±0.81^b^41.62±1.85	^a^42.93±3.54^b^44.55±1.05
**GGT**(U/L)	^a^32.45±2.78^b^36.10±2.91	^a^37.52±8.84^b^26.07±2.38	^a^40.23±16.25^b^23.55±4.95
**ALP**(U/L)	^a^131.1±41.51^b^83.83±34.59	^a^82.28±20.54^b^77.82±07.14	^a^146.6±34.20^b^90.30±17.10
**TBA**(μmol/L)	^a^10.20±2.27*^b^3.23±0.69	^a^8.28±1.70^b^4.75±1.48	^a^5.80±1.75^b^7.05±3.65
**GLU**(mmol/L)	^a^7.32±0.60^b^6.77±0.26	^a^8.38±0.77^b^7.07±0.41	^a^7.22±0.56^b^6.06±0.04
**BUN**(mmol/L)	^a^18.33±3.78^b^19.64±0.77	^a^19.04±1.15^b^17.66±1.26	^a^11.84±5.44^b^21.10±1.78
**Co2**(mmol/L)	^a^22.80±0.94**^b^12.47±3.18	^a^23.90±0.98***^b^17.28±1.24	^a^25.43±0.46**^b^19.25±0.25
**UA**(μmol/L)	^a^35.80±7.40^b^32.27±4.16	^a^40.67±5.70^b^46.22±8.80	^a^40.27±5.01^b^58.15±18.35
**TC**(mmol/L)	^a^5.66±0.49^b^4.41±0.49	^a^6.57±0.72^b^7.23±2.23	^a^5.66±0.21^b^8.63±3.41
**TG**(mmol/L)	^a^0.92±0.92^b^2.52±1.38	^a^1.16±0.09^b^1.16±0.32	^a^1.20±0.18^b^3.32±0.97*
**HDL-c**(mmol/L)	^a^2.39±0.24*^b^1.77±0.08	^a^2.56±0.15^b^2.29±0.34	^a^2.60±0.07**^b^2.14±0.01
**LDL-c**(mmol/L)	^a^0.45±0.05^b^0.61±0.27	^a^0.52±0.07^b^0.80±0.38	^a^0.99±0.54^b^0.78±0.09
**CK**(U/L)	^a^59.35±8.91^b^51.13±15.92	^a^129.5±36.17*^b^51.98±5.82	^a^106.8±28.67^b^25.60±22.90
**LDH**(U/L)	^a^140.0±7.24^b^230.4±54.87	^a^159.8±24.04^b^297.7±58.95*	^a^97.70±26.13^b^237.3±55.90*
**AMS**(U/L)	^a^18.60±2.65^b^135.7±119.5	^a^16.72±1.38^b^31.02±8.81	^a^16.57±0.32***^b^2.35±0.05
**Lipase**(U/L)	^a^10.78±0.48^b^10.63±0.08	^a^12.15±0.61^b^11.44±0.68	^a^10.83±0.29^b^9.95±0.65
**Cr**(μmol/L)	^a^78.40±15.49^b^69.30±15.99	^a^53.82±4.78^b^59.13±1.70	^a^51.97±6.56^b^68.90±7.60
**K**(mmol/L)	^a^3.97±0.10^b^4.22±0.30	^a^4.32±0.22^b^4.49±0.13	^a^4.24±0.40^b^4.67±0.01
**Na**(mmol/L)	^a^156.6±3.00^b^163.2±0.97	^a^156.6±0.85^b^157.9±1.07	^a^153.5±2.43^b^162.6±0.95*
**Cl**(mmol/L)	^a^109.8±0.67^b^109.8±1.10	^a^105.0±1.21^b^107.8±1.29	^a^106.4±1.45^b^111.4±2.70
**Ca**(mmol/L)	^a^2.47±0.02^b^2.73±0.09*	^a^2.42±0.03^b^2.41±0.07	^a^2.51±0.04^b^2.44±0.16
**P**(mmol/L)	^a^0.91±0.21^b^1.75±0.29*	^a^0.97±0.11^b^1.54±0.09**	^a^0.89±0.24^b^1.84±0.15*

^(a)^ = Tina-E-Zhou Population,

^(b)^ = Poyang Lake population.

Significant difference with *P<0.05, **P<0.01 and ***P<0.0001.

**Table 5:** Hematologic parameters of reproductive states groups.

Parameters	Pregnant (Mean±SEM)	Lactating (Mean±SEM)	Pregnant + Lactating (Mean±SEM)
**RBCs**(x 10^12^/L)	^a^5.13±0.11*^b^4.62±0.07	^a^5.41±0.20*^b^4.83±0.07	^a^5.39±0.313^b^4.84±0.060
**Hb** (g/L)	^a^167.3±1.10*^b^149.3±6.88	^a^175.0±4.29**^b^149.0±6.24	^a^175.3±6.52*^b^139.0±9.00
**HCT**(%)	^a^41.94±12.16^b^43.60±1.60	^a^56.56±1.43**^b^37.51±7.43	^a^55.53±2.78*^b^21.55±21.05
**MCH**(pg)	^a^32.63±0.67^b^32.33±1.13	^a^32.44±0.52^b^30.78±0.93	^a^32.65±0.85^b^28.75±2.25
**MCHC**(g/L)	^a^308.5±1.19^b^342.7±9.06**	^a^309.4±2.47^b^331.3±13.98	^a^316.0±2.48^b^305.0±43.0
**MCV**(fL)	^a^105.8±2.38*^b^94.37±3.52	^a^104.9±1.43***^b^93.28±1.79	^a^103.3±1.88^b^95.25±6.15
**PLT**(x 10^9^/L)	^a^122.5±13.77^b^98.0±2.08	^a^148.3±14.88^b^146.8±34.22	^a^152.8±12.57^b^112.0±15.00
**MPV**(fL)	^a^11.93±0.30^b^12.30±0.61	^a^11.50±0.28^b^12.90±0.75*	^a^11.33±0.04^b^13.75±2.45
**PCT**(%)	^a^0.14±0.01^b^0.12±0.00	^a^0.16±0.01*^b^0.13±0.00	^a^0.17±0.01^b^0.14±0.00
**PDW10** (GSD)	^a^54.60±38.13^b^16.07±0.12	^a^16.60±0.18^b^16.58±0.51	^a^16.15±0.050^b^19.05±2.95
**RDW**(% cv)	^a^13.10±0.35^b^13.40±0.17	^a^12.56±0.19^b^12.90±0.31	^a^13.13±0.23^b^13.95±0.25
**WBCs**(x 10^9^/L)	^a^1.61±0.12^b^2.08±0.20* ^Log^	^a^6.81±0.41^b^5.66±0.64	^a^5.36±0.76^b^5.00±0.20
**Neutrophil**(%)	^a^73.10±3.81^b^63.70±10.12	^a^75.86±1.71^b^58.93±12.62	^a^62.68±5.03^b^60.95±0.05
**Lymphocyte**(%)	^a^20.60±2.50^b^23.37±7.97	^a^16.99±1.34^b^24.38±2.41**	^a^29.55±4.29^b^24.95±0.25
**Monocyte**(%)	^a^0.85±0.29^b^1.86±0.08*	^a^1.48±0.23^b^12.50±11.07	^a^1.17±0.36^b^2.40±0.30
**Basophil**(%)	^a^0.05±0.02^b^0.07±0.02	^a^0.07±0.01^b^0.07±0.02	^a^0.10±0.04^b^0.27±0.02*
**Eosinophil**(%)	^a^5.40±2.01^b^10.97±2.32	^a^5.60±1.35^b^6.00±2.15	^a^6.50±1.96^b^10.45±0.45

^(a)^ = Tina-E-Zhou Population,

^(b)^ = Poyang Lake population.

Significant difference with *P<0.05, **P<0.01 and ***P<0.0001.

### Lactating females (LF)

The results showed a statistically significantly higher serum level of ALT, AST, CK and CO_2_ in LF living in the Tian-E-Zhou Oxbow, while TBILI, LDH and P levels were statistically significantly higher in YFPs living in Poyang Lake (Table 4). LF living in the Tian-E-Zhou Oxbow had statistically significantly higher levels of RBCs, HB, HCT, MCV and PCT while porpoises living in Poyang Lake had statistically significantly higher levels of MPV and lymphocytes (Table 5).

### Pregnant plus lactating (PL)

The results showed statistically significantly higher serum levels of HDL-c, AMS and CO_2_ in the Tian-E-Zhou Oxbow PL group as compared to Poyang Lake group. However, statistically significantly higher serum levels of TBILI (log transformed), TBILb, DBILb, LDH, Na, P and TG were observed in porpoises living in Poyang Lake (Table 4). YFPs living in the Tian-E-Zhou Oxbow, showed statistically significantly higher Hb and HCT levels. Only the basophil count was statistically significantly higher in YFPs living in Poyang Lake (Table 5).

### Results across the groups of Tina-E-Zhou population

**Fig 1:**
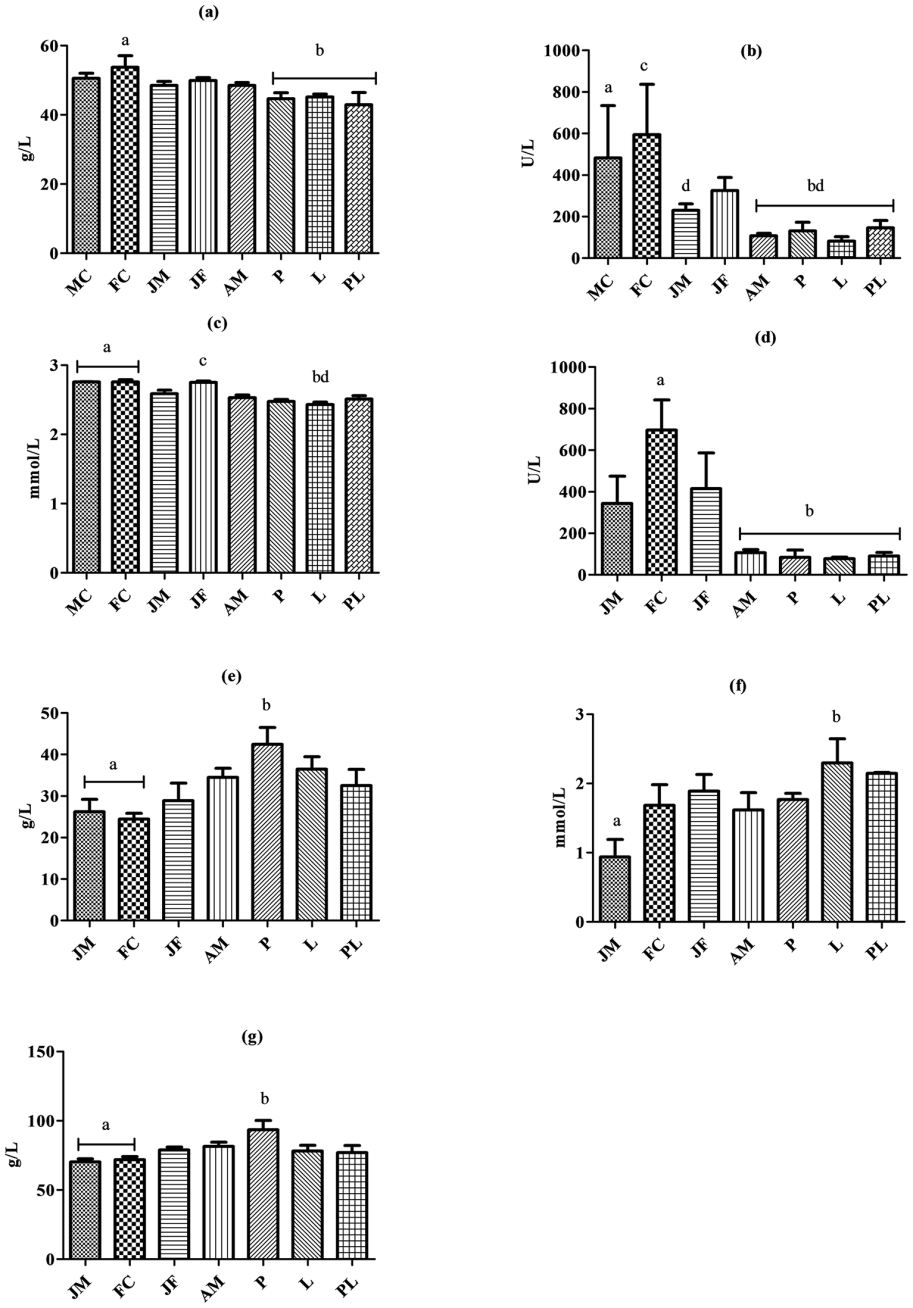
Biochemical parameters across the groups. YFPs living in the Tian-E-Zhou Oxbow (a) serum albumin, (b) ALP and (c) Ca. YFPs living in Poyang Lake (d) ALP, (e) GLB, (f) HDL-c and (g) TP levels. Biochemical parameter in each population followed by the same letter was not significantly different at *P* = 0.05.

For YFPs living in the Tian-E-Zhou Oxbow, a one-way ANOVA showed that the serum albumin in female YFPs decreased statistically significantly across the various reproductive states. The FC group showed a statistically significantly higher serum albumin versus the P, the L and the PL groups. Serum ALP showed a statistically significant decreased across the ontogenetic and reproductive states and was significantly higher in the MC versus the AM, the P, the L and the PL groups. Similarly, members of the FC group showed a statistically significantly higher serum ALP levels compared to JM, AM, P, L and PL (Fig 1). We observed a statistically significant decline in Ca levels across the reproductive states in females. Both FC and JF showed a statistically significantly higher Ca level compared to the L group (Fig 1).

**Fig 2:**
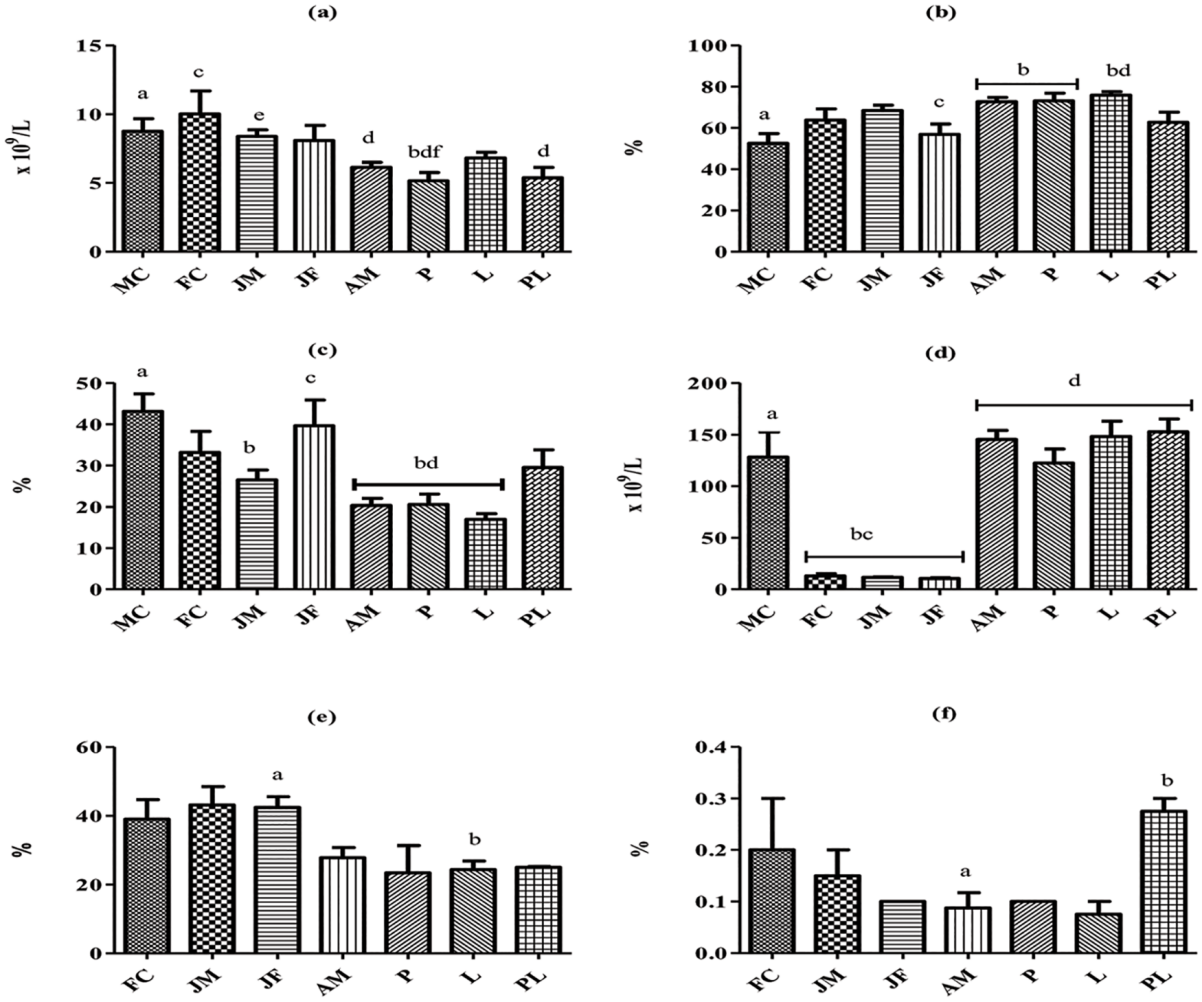
Blood parameters across the groups. YFPs living in the Tian-E-Zhou Oxbow (a) WBC, (b) neutrophil (c) lymphocyte and (d) PLT count. YFPs living in Poyang Lake (e) lymphocyte and (f) basophils count. Blood parameter in each population followed by the same letter was not significantly different at *P* = 0.05.

A one-Way ANOVA of hematology across the groups showed statistically significantly higher lymphocyte counts in MC, and JF and WBC counts in both FC and JM, compared to the higher ontogenetic and reproductive states. Neutrophil counts were higher in AM, P and L group vs. MC and L vs. JF. PLT counts across the groups showed significantly higher value in MC vs. Juvenile states. However, in comparison with FC, JM and JF, an age and a reproductive status dependent increase was observed in the AM, P, L and PL groups (Fig 2).

### Results across the groups of Poyang Lake population

For YFPs living in Poyang Lake, serum ALP was statistically significantly higher in FC compared to AM, P, L and PL groups. Serum GLB was statistically significantly higher in P versus FC and JM. Similarly, serum HDL-c was statistically significantly higher in L versus JM group. In the P group, serum TP was statistically significantly higher compared to JM and FC groups (Fig 1). Across all the groups, basophil counts were statistically significantly higher in PL versus AM and lymphocytes were statistically significantly higher in JF versus L group (Fig 2).

## Discussion

### Lipid profile

**Fig 3:**
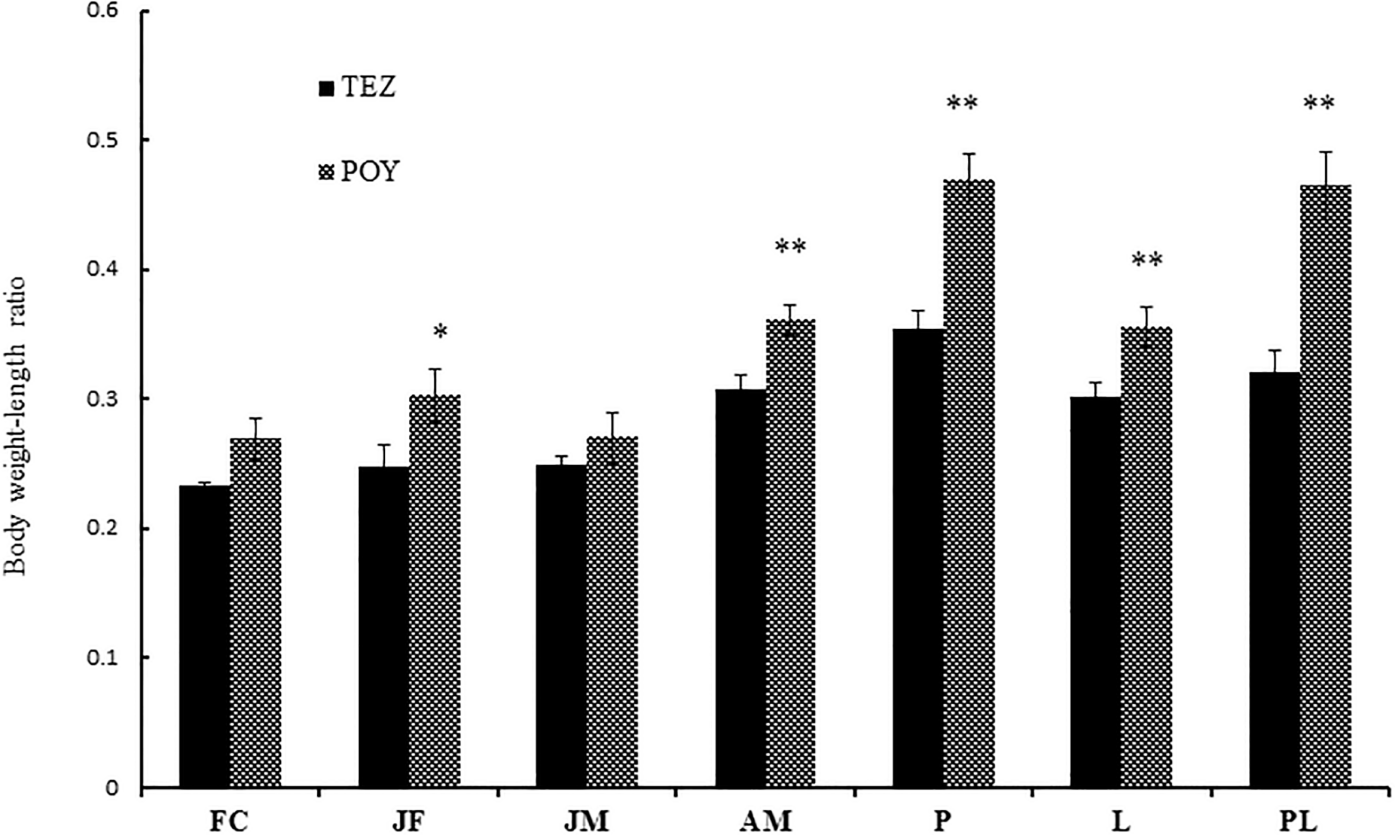
Comparison of body weight/length ratio between the Poyang Lake and Tian-E-Zhou Oxbow YFPs.

In the present study, we found significantly higher serum levels of TC, HDL-c and lipase in the YFPs living in the Tian-E-Zhou Oxbow. Significantly higher levels of TG as well as LDL-c were detected in YFPs living in Poyang Lake. Triacylglycerols as compared to squalene, diacyl glyceryl ethers and wax esters have a minimum role in buoyancy. However, the main function of triacylglycerols is energy storage which can be utilized for energetically exhausting activities such as reproduction, migration, or during prolonged periods of low prey availability [34,35]. Variations in organism energetic profiles depend on the diet at different temporal scales [36] and can be directly linked with habitat utilization, reproductive cycle and nutritional conditions [37,38]. St. Aubin and Geraci (1989) observed in captive beluga whales (*Delphinapterus leucas*) higher levels of cholesterol and triglyceride within 10 weeks after feeding on oil-rich herring rather than their normal diet of decapod crustaceans [39]. Higher cholesterol levels have also been reported in captive beluga whales, bottlenose dolphins (*Tursiops truncates*) and free-ranging pantropical spotted dolphins (*Stenella attenuata*), suggesting the positive effects of diet and environment on the lipid profile of animals [40–42]. In general, marine mammals have elevated cholesterol levels which may reflect the high cholesterol content of their diet. During fasting conditions, intestinal and hepatic cholesterol synthesis is either absent or significantly reduced and the hypercholesterolemia related to food scarcity seen in different species that are not adapted to long-term fasting is illustrative of cholesterol mobilization from adipose tissue [43,44]. A recent study in fasting adult male elephant seals (*Mirounga angustirostris*) revealed highly elevated cholesterol levels that declined with adipose tissue stores across the fast [45]. These animals were able to maintain elevated high-density lipoprotein (HDL) levels despite dramatic reductions in total cholesterol and low-density lipoproteins (LDL) associated with fasting. This selective depletion of serum LDL across the fast suggests the metabolic adaptation to fasting may include alterations in cholesterol metabolism. In YFPs living in Poyang Lake, we observed a statistically significantly higher body weight/body length ratio in JF, AM, P, L and PL groups. However, in FC and JM, the body weight/body length ratio was not statistically significantly higher, but was greater in the Poyang Lake YFPs (Fig 3). Body weight/length ratio is the most appropriate indicator for evaluating the status of an individual or a population [46,47]. The significantly lower body weight/length ratio and the significantly higher lipid profile for porpoises living in the Tian-E-Zhou Oxbow indicate a poor nutritional status.

### Hepatic enzymes

We observed significantly elevated serum levels of AST and ALT in all the groups living at the Tian-E-Zhou Oxbow, except in P and PL animals. However, the various fractions of serum bilirubin were significantly elevated in many groups living in Poyang Lake. Elevated serum bilirubin values in Poyang Lake porpoise populations may indicate increased RBC breakdown. We observed significantly lower RBCs count, liver dysfunction, obstructive liver diseases, and other health problems in this population [48,49]. Though acute stress in phocids, resulting from handling, apprehension, excitement and fear can cause splenic contraction when adrenaline is released, resulting in an increase of circulating erythrocytes [50,51]. However, we did use the same capture methods for both populations. In cetaceans (whales, porpoises and dolphins) liver diseases have been frequently reported [52] and the etiologies include trematodes [53], sarcocystis [54], suspected acquired immunodeficiencies [55] and a hepatitis B–like virus [56]. These causes may occur in succession or concurrently [57] elevating serum ALT and AST levels [58]. Venn-Watson et al. (2008) reported an episodic increase in serum ALT and AST over a 9 year period, which was later identified as chronic hepatitis with excessive iron deposition [59]. Like our study, Venn-Watson et al. (2008) further reported an episodic increase in AST and ALT with higher serum cholesterol (observed), and GGT, suggesting steatosis [59,60] and cholestasis including, autoimmune hepatitis, sclerosing cholangitis and primary biliary cirrhosis [61,62]. Apparently, in Tian-E-Zhou Oxbow, the water quality was worse than Poyang Lake. The Oxbow is not only fed by the Yangtze River but also by run offs from surrounding fields and marshes in which pesticides, excess nutrients and other pollutants are added. Therefore, the elevated levels of ALT and AST in Tian-E-Zhou population could be due to the persistent exposure to these various pollutants. A number of studies reported elevated levels of ALT and AST in aquatic mammals as well as in humans in response to pollutants [63–66]. The epidemiological, clinical and biochemical evidence presented so far indicate that ALT may be crucial as a screening test for the early detection of asymptomatic liver disease and probably for other etiologies related to premature mortality [67,68]. Measuring ALT may be a useful screening tool for the detection of chronic liver disease, which may ultimately lead to hepatocellular carcinoma and/or end-stage liver disease and liver cirrhosis. Similar to the results found in *Phocoena phocoena* [69] and in humans [70], we also observed a significantly higher serum ALP level in both populations during the early life stages in both groups. Therefore, ALP levels could be used as an indicator of physical maturity in porpoises and in other mammals [69].

### Enzymes, electrolytes and other biochemical parameters

An elevation in serum CK levels is normally linked to skeletal or cardiac muscle injuries, although a rise in CK levels is also associated with an animal struggling or being captured [71]. The higher levels found in the porpoises living in the Tian-E-Zhou Oxbow may, therefore, be a result of increased stress and chasing, as they are rarely exposed to anthropogenic activities compared to the porpoises living in Poyang Lake. Although we used the same capture procedure, still the animals living in Poyang Lake seem calmer when they were circled by the net, while the animals living in the Tian-E-Zhou Oxbow seem to be very active; suggesting that their elevated CK levels could be due to hyper-muscular activities [72]. Further, higher serum CK is also linked to the catabolism of skeletal muscles, resulting from starvation which is also reflected by a lower serum level of creatinine [73] which we observed in the Tiana-E-Zhou Oxbow porpoise population. However, elevated levels of serum LDH and creatinine in Poyang Lake porpoise population could also be related to rhabdomyolysis associated with capture stress. This may also occur through prolong physiologic exertion changes [74], as Poyang Lake is a vast lake.

Plasma protein and BUN values are influenced by the hydration state of mammals, diet, age, activity, reproductive status, and environmental conditions [75–77]. These values are used as an indicator of starvation or fasting in adult marine mammals [78]. When compared to porpoises living in Poyang Lake, porpoises living in the Tian-E-Zhou Oxbow exhibited elevated plasma BUN and protein levels in early life stages. For the management of wild animals, BUN levels are mostly used as a hematological indicator of nutritional stress [79]. The mechanism is that, when glucose is exhausted, animals turn to adipose tissue as their principal energy source [80], enhancing lipolysis and reducing BUN levels [81]. In the case of extended fasting, animals shift from lipolysis to increased body protein catabolism for energy [82], causing elevated BUN levels [81]. As compared to food-restricted animals, BUN levels in fasting animals are more difficult to analyze, as concentrations of BUN are the net result of both tissue catabolism and food protein digestion (including feeding frequency, protein quality and quantity) [83]. In harp seal (*Phoca groenlandica*) pups, Worthy (1982) observed no clear differences in circulating levels of protein and BUN during a post-weaning fast when compared with the onset of feeding [84] indicating a variation in species-specific response towards the stages of fasting [85]. Elevated serum P levels in porpoises living in Poyang Lake could be associated with excess dietary phosphorous, rhabdomyolysis, and hemolysis or decreased glomerular filtration rate [49]. During pregnancy and lactation, serum Ca levels fell due to a rapid requirement for fetal skeletal mineralization and for nursing infants along with a decrease in serum albumin due to hemodilution [86]. However, for porpoises living in Poyang Lake, both TP and GLB were significantly higher in P versus JM and FC, suggesting the effects of pregnancy.

### Hematological parameters

Anemia in marine mammals is characterized by a clinically significant decrease in RBC, Hb and HCT concentrations. Many factors can cause anemia including, low-grade blood loss, pregnancy, poor nutritional status, liver diseases and chronic disease/inflammation. Over hydration can also result in anemia while dehydration can mask anemia. Therefore, anemia has different classifications based on the disease etiology [49]. Anemia due to a chronic disease in dolphins has been associated with a decrease in albumin [49] and an increase in bilirubin [87]. This was observed in porpoises living in Poyang Lake. However, significantly higher MCHC levels, which could have resulted in dehydration, complicated our study. Parasitic infections are another cause of anemia in cetaceans. A total of 13 helminthic species (1 acanthocephalan, 2 cestodes, 4 trematodes and 6 nematodes) are reported to infect *N*. *asiaeorientalis* [88] as well as *Neophocaena asiaeorientalis ssp*. *Asiaeorientalis*. Necropsy has resulted from this type of infection (unpublished data). Further research into the etiology of anemia is needed to determine the type of anemia present in a population.

For stress and inflammation, the most common indicators used are lymphocyte and WBCs counts. The significantly higher count of WBCs, lymphocytes and monocytes observed in porpoises living in Poyang Lake suggest a chronic exposure to infections, especially parasites and viruses. Chronic exposure to infections have been reported in bottlenose dolphins [41,89,90]. Similarly, to YFPs living in Poyang Lake, higher eosinophil counts were also observed in bottlenose dolphins [41] and wild harbor porpoises [91] indicating heavy parasites infestations in their natural environment [92]. Higher basophils are rarely seen in marine mammals with unclear significance [90]. However, higher neutrophil counts were observed in the porpoise population living in the Tian-E-Zhou Oxbow which may indicate bacterial or fungal infections [89,90]. In terms of lymphocyte and WBCs counts, age class and reproductive states were a significant variable: both were significantly higher in either calves or juveniles compared to adults in both populations. Similar results have been reported in harbor seals [93], sheep, cows, dogs [94] and buffalo [95]. Contrary to lymphocytes count, neutrophil count was higher in AM, P and L stages across the groups for porpoises living in the Tian-E-Zhou Oxbow. Similar results were observed in buffalo. However, higher neutrophil counts in lactating animals could be due to lactational stress leading to the release of endogenous corticosteroids [95]. In a Atlantic bottlenose dolphins, the platelet count decreases significantly with increasing age [96]. Similar results were found in MC compared to JM and JF porpoises living in the Tian-E-Zhou Oxbow. However, the reason for higher platelets counts in AM, P, L and PL porpoises living in the Tian-E-Zhou Oxbow is unknown. The higher count could depend on the genetic, environmental and physiological health status of the animals [97].

### Conclusions and future recommendations

In summary, due to variation in habitat quality, we found significant variations in important physiological parameters between two populations. Porpoises living in the Tian-E-Zhou Oxbow seemed malnourished compared to the porpoises living in Poyang Lake. There was a significantly lowered body weight/length ratio and higher BUN and lipid profile in porpoises living in the Tian-E-Zhou Oxbow. Furthermore, the apparently poor water quality of the Tian-E-Zhou Oxbow was also reflected by significantly higher hepatic enzymes such as ALT and AST in the porpoises. This suggested hepatic dysfunction. In the Poyang Lake porpoise population, significantly higher count of WBCs, lymphocytes, monocytes and eosinophils indicated chronic exposure to infections, especially parasites and viruses, while a higher neutrophil count in the Tiana-Zhou Oxbow porpoise population suggested bacterial or fungal infections. Further, the higher level of CK in the Tian-E-Zhou Oxbow population could be due to hyper-muscular activity and the higher LDH and creatinine levels in porpoises living in Poyang Lake indicated either rhabdomyolysis or prolong physiological exertions. Elevated serum P levels in porpoises living in Poyang Lake could be associated with excess dietary phosphorous, rhabdomyolysis, and hemolysis or a decreased glomerular filtration rate. This study will encourage clinicians to further investigate hematology and biochemical parameters of porpoises living under free-ranging and semi-natural conditions. Understanding the correlation between age, sex, health status, and location will improve study designs for future health assessment studies of these animals. Further, these values are going to be of explicit use for the interpretation of hematology and clinical chemistry values measured in YFPs suspected of chronic or acute diseases. Furthermore, these values will be used for the interpretation of hematology and clinical chemistry data measured in YFPs suspected of chronic or acute diseases. Furthermore, the use of metabolomics, genomics and proteomics are needed for the health assessment of aquatic mammals, instead of simply relying on conventional serum chemistry and hematology for a health assessment. Based on our study, we also suggest an increased fish stock and improved water quality at the Tian-E-Zhou Oxbow. However, at Poyang Lake, we recommend minimizing human interference.
